# Supplementary material for: Electronic Structure and Solvation Effects from Core and Valence Photoelectron Spectroscopy of Serum Albumin
Source: Int J Mol Sci. 2022 Jul 26;23(15):8227. doi: 10.3390/ijms23158227 (PMC9331649; doi:10.3390/ijms23158227)
Supplement: Supplementary file 1 [file ijms-23-08227-s001.zip › ijms-1798126-supplementary.pdf]

Supplementary Information for
Electronic structure and solvation effects from core and valence photoelectron spectroscopy of serum albumin

Jean-Philippe Renault*, Lucie Huart, Aleksandar R. Milosavljević, John D. Bozek, Jerôme Palaudoux, Jean-Michel Guigner, Laurent Marichal, Jocelyne Leroy, Frank Wien, Marie-Anne Hervé Du Penhoat, Christophe Nicolas*

*Jean-Philippe Renault and Christophe Nicolas
Email: jean-philippe.renault@cea.fr and Christophe.nicolas@synchrotron-soleil.fr

This file includes:

Figures S1 to S9
Tables S1 to S3
SI References

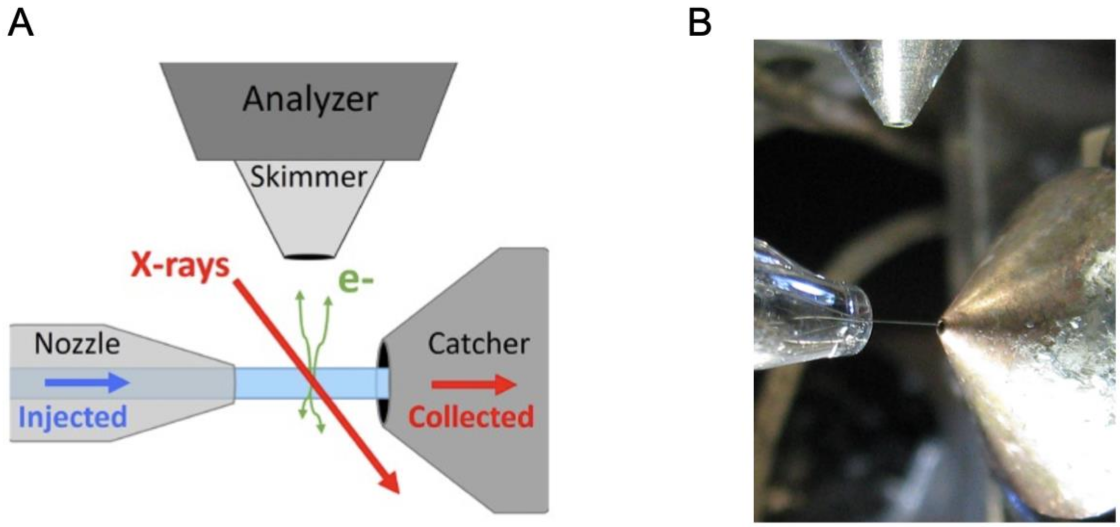

Figure S1. (A) Scheme of the liquid micro-jet experiment showing the injected liquid (blue), synchrotron radiation beam (red), and ejected electrons (green). (B) Picture of the liquid jet in the lowest position compared to the electron skimmer. During measurement the liquid jet is at around 1.5 mm from the electron skimmer.

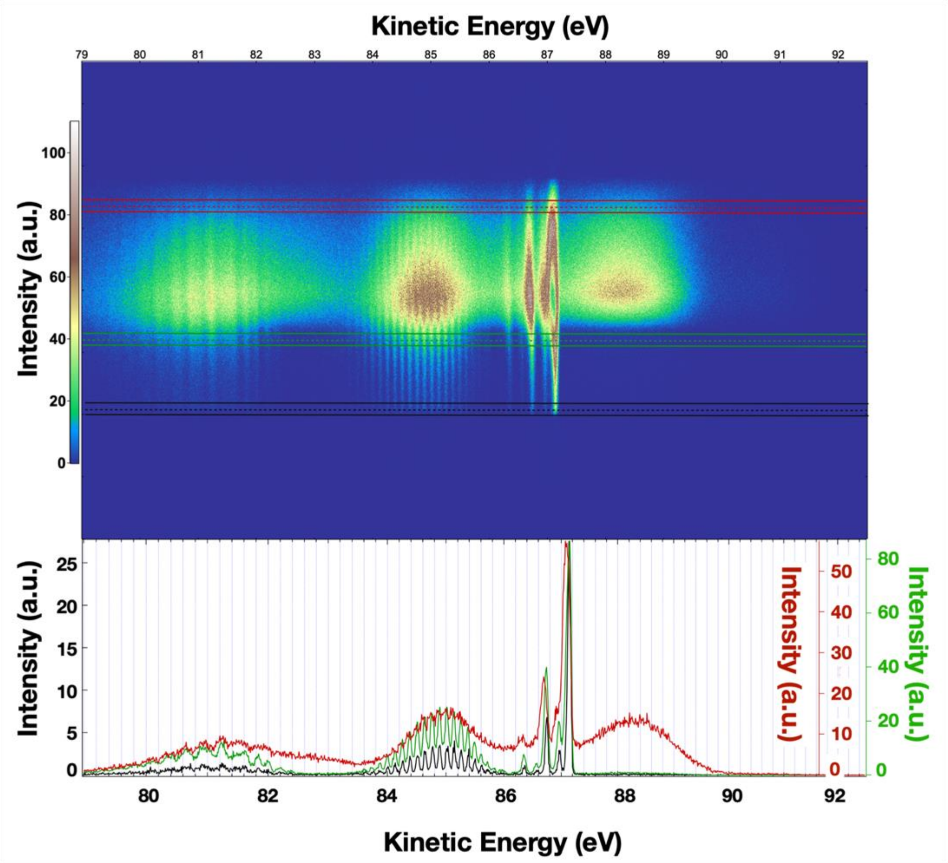

Figure S2. Recent publications demonstrate the challenge of accurately measuring the ionization energy of liquid water [25,78]. We propose a new approach to reference our energies compared to the vacuum level. For this we use the spatial information given by the position sensitive detector of the VG-Scienta spectrometer. The top panel displays a false color map of a valence band photoelectron spectrum recorded at 100 eV and in the transmission mode of the analyzer. The kinetic energies of the electrons are dispersed on the X-axis, and the Y-axis represents the non-energy axis. By integrating (between the two continuous lines from a same color) only one part of the image far from the center (far from the jet contribution), it is possible to retrieve a “pure” gas phase spectrum (black line on the bottom panel), which then can be used via the literature to calibrate the ionization energies compared to the vacuum level at infinity of the X 1B1 gas phase state. Closer to the center of the color map, the different vibration lines of the different gas phase electronic states start to broaden and to shift towards lower kinetic energies, due to the field perturbation induced by the liquid jet (green curve of the bottom panel). To reach a sufficient signal for the BSA valence bands, the peak of the X 1B1, had to be saturated (too many counts/s for the detector, which operates in counting mode). Note that the saturation does not affect the black spectrum. The ionization energy of the 1b1 orbital can be shifted up to ~0.1 eV, as shown in the red curve of the bottom panel. This 0.1 eV difference should be the order of magnitude in energy difference between the vacuum level at infinity and the vacuum level at the surface (the electrons still feel the field of the surface from which they are emitted).

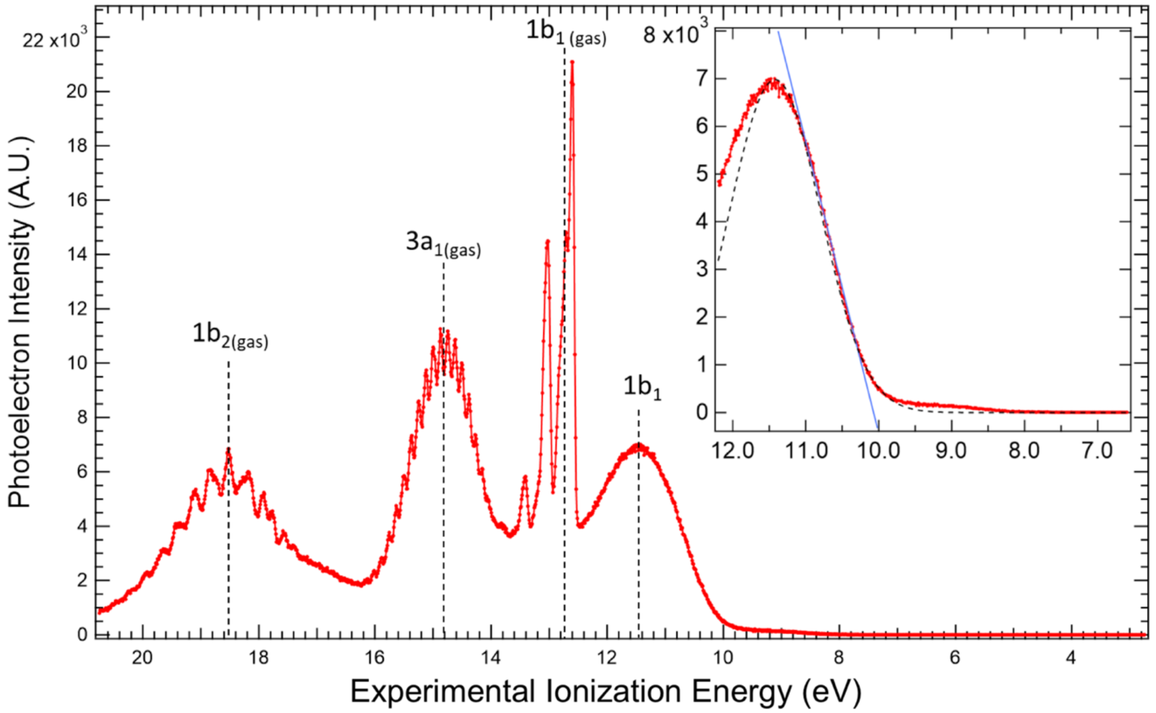

Figure S3. Valence photoelectron emitted from the liquid phase of the BSA aqueous solution (36 g/L) measured at 100 eV photon energy. Peaks due to ionization of the three valence orbitals of water gas phase water are labeled as well as the liquid phase peak. The liquid water contribution of $1b_1$ molecular orbital was fit with a Gaussian function. Resulting fitting parameters define the $1b_1$ signal centered at 11.4 eV with a full width at half maximum (FHWM) of 1.76 eV. The photoionization threshold is determined by a linear extrapolation of the slope of the $1b_1$ signal (inset, blue line) and gives a value of 10.0 eV.

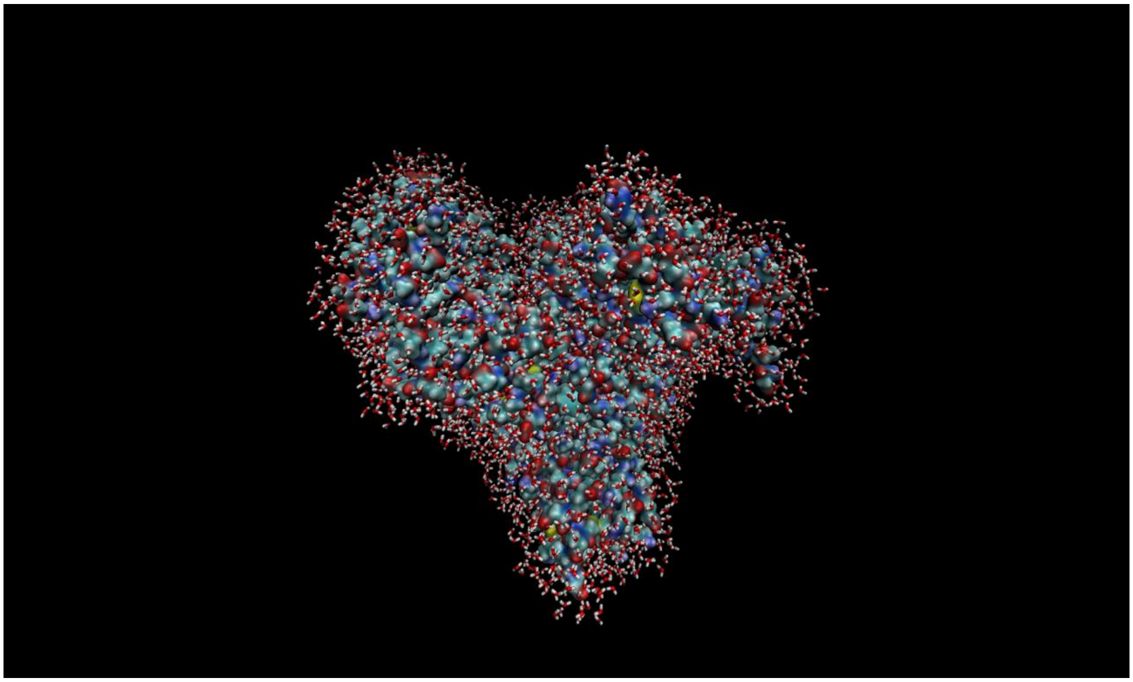

Figure S4. Bovine serum albumin structure in heart-shape with a 3 Å water added layer.

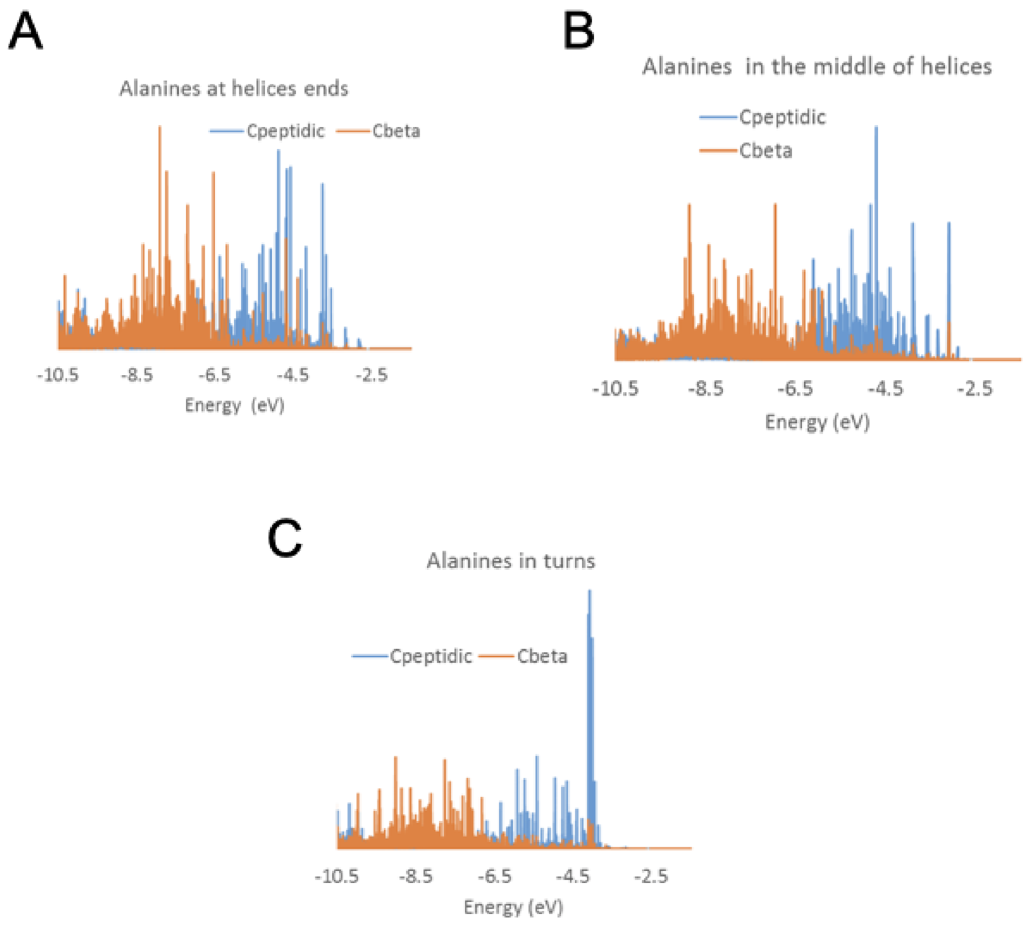

Figure S5. Local density of states for peptidic and beta carbons of alanines, in various secondary structures (A) at the helix end, (B) in the middle of the helix and (C) in turns.

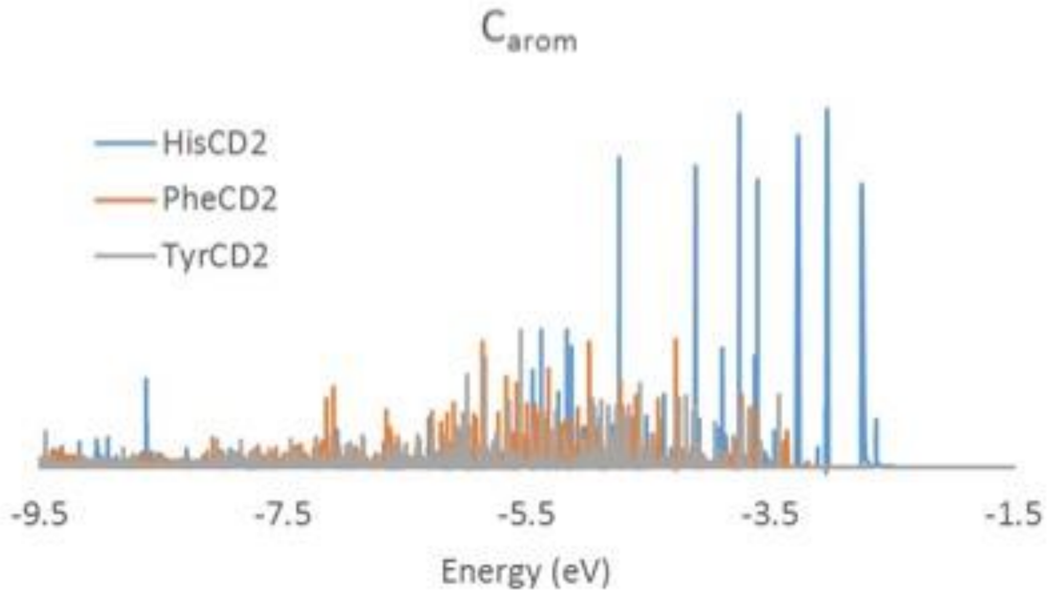

Figure S6. Local density of states, in the valence region, for aromatic carbons in various secondary structures.

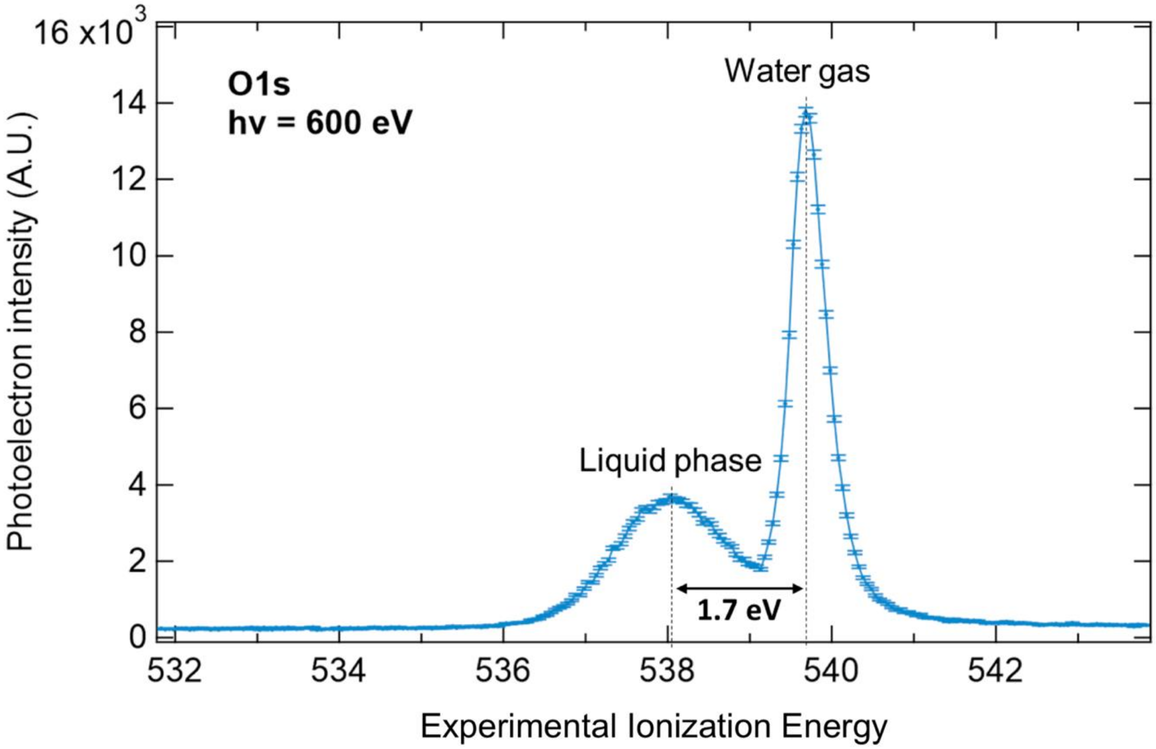

Figure S7. Oxygen XPS spectra of the bovine serum albumin (BSA) recorded at 600eV in the liquid micro-jet. Water gas signal is centered as 539.8 ± 0.2 eV while the liquid phase is centered at 538.1 ± 0.2 eV.

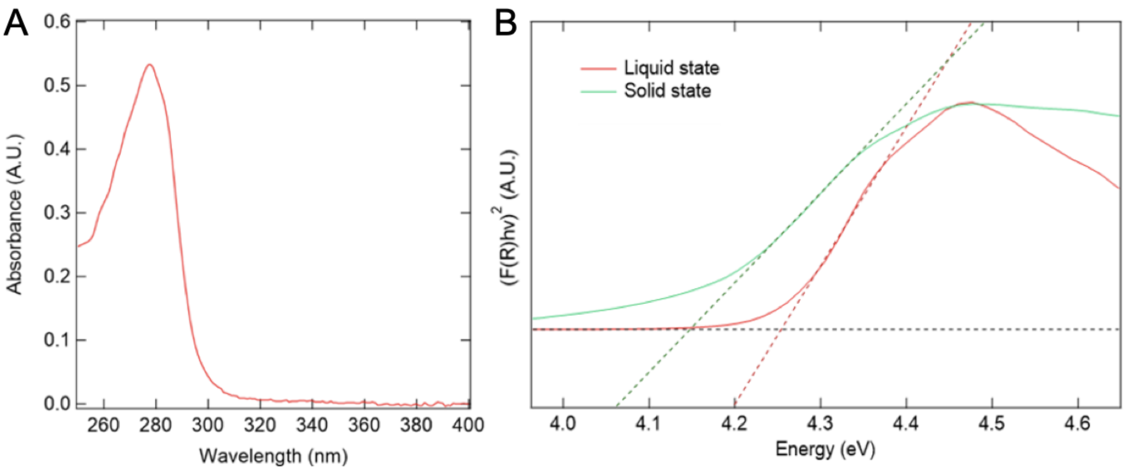

Figure S8. According to Tauc, the band gap energy in semi-conductors can be determined following the dependence of the absorption coefficient (α) on the photon energy (hv) for near-edge optical absorption using the following equation [79]:

$$(\alpha h\nu)^{\frac{1}{2}} = B(h\nu - E_g) \qquad \textit{Equation S1}$$

where E_g is band gap energy.

(A) Optical absorption spectra of the BSA solution (1 g/L in pure water) and (b) Tauc plot ($(\alpha hv)^{1/2}$ versus hv plot) for the BSA solution (red) and BSA crystals (green). Linear regions were extrapolated to the energy axis. (B) The value of the band gap energy measured is respectively E_g^{liquid}= 4.25±0.07 eV and E_g^{solid}= 4.15±0.08 eV.

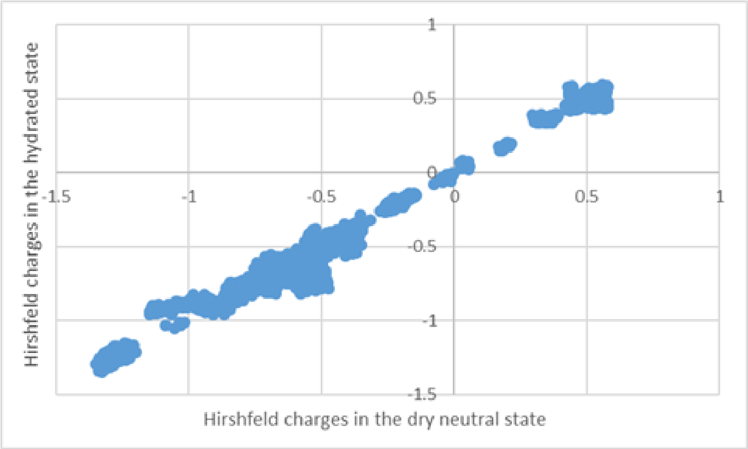

Figure S9. Comparison of the Hirschfeld charge calculations with and without water added. The slope close to one confirms that hydration induces limited changes in charges.

Table S1. Secondary structural content of BSA assessed by deconvolution of far-UV (165-260nm) SRCD spectra using BeStSel web server.

Treatment	α-helix	β-elements	Others
**Before Injection**	72.4	8.0	19.7
**After Collection**	73.5	8.1	18.5

Table S2. XPS spectral features of bovine serum albumin from the literature.

Ref	C1S (eV)				N1s (eV)		Calibration	
	C-C/C-H	C-O / C-N	C=N/N C=C/O-C-O	OC=O N-C=O				
[80]	285±0.5	286.6±0.5		288.4±0.5	400±1 (organic)		C-H at 285 eV	
[81]	286.5±0.2	287.4±0.2		289.4±0.2	401.3±0.2		Ag 3d$_{5/2}$	
[82]	285	286.2	287.5	288.3	400		C-H at 285 eV	
[83]	284.6	285.8		287.6	400		C-H at 285 eV	
[84]	284.8	286.1		288.2	400.2		C-H at 284.8 eV	
[85]	285	286.4		288.3	400.2		C-H 285 eV	
[86]			287.9±0.4	289±0.3	400.0±0.2	401.4±0.2	Au 4f$_{7/2}$,Cu 2p$_{3/2}$, and Ag 3d$_{5/2}$ *	
[87]	285.4 ±0.2	286.5 ±0.2	287.8 ±0.2	288.8±0.2	400.9		Au 4f$_{7/2}$,Cu 2p$_{3/2}$*	
[88]	285±0.5	286.6±0.5		288.4±0.5	NH 399	NH_3^+ 401	C-H at 285 eV	
[89]	285.4	286.7	287.9	288.7	400.8		Au 4f$_{7/2}$,Cu 2p$_{3/2}$*	
[90]	284.7	286	287.7		C=N 397.5	C- N 400	C-H at 285 eV	
[10]	285	286.2	287.4	288.6	400	401.2	402.4	C-H at 285 eV

Table S3. Distance of various carbons with respect to the protein surface. The distances were calculated using Biopython and MSMS [91]

Carbon Type	$C_{peptidic}$	C_{beta}	C_{gamma}	C_{delta}
Average distance from the surface (Å)	2.58	2.50	2.52	2.51

SI References

10. Jain, V.; Kjærvik, M.; Bahr, S.; Dietrich, P.; Meyer, M.; Thißen, A.; Linford, M.R. Bovine serum albumin, aqueous solution, by near-ambient pressure XPS. *Surf. Sci. Spectra* **2019**, *26*, 014027.
25. Thürmer, S.; Malerz, S.; Trinter, F.; Hergenhahn, U.; Lee, C.; Neumark, D.M.; Meijer, G.; Winter, B.; Wilkinson, L. Accurate Vertical Ionization Energy and Work Function Determinations of Liquid Water and Aqueous Solutions. *Chem. Sci*. **2021**, *12*, 10558–10582.
78. Pérez Ramírez, L.; Boucly, A.; Saudrais, F.; Bournel, F.; Gallet, J.J.; Maisonhaute, E.; Milosavljević, A.R.; Nicolas, C.; Rochet, F. The Fermi Level as an Energy Reference in Liquid Jet X-Ray Photoelectron Spectroscopy Studies of Aqueous Solutions. *Physical Chemistry Chemical Physics* **2021**, *23*, 16224–16233, doi:10.1039/d1cp01511g.
79. Tauc, J.; Grigorovici, R.; Vancu, A. Optical Properties and Electronic Structure of Amorphous Germanium. *physica status solidi (b)* **1966**, *15*, 627–637, doi:10.1002/pssb.19660150224.
80. Muñoz, A.I.; Mischler, S. Electrochimica Acta Electrochemical Quartz Crystal Microbalance and X-Ray Photoelectron Spectroscopy Study of Cathodic Reactions in Bovine Serum Albumin Containing Solutions on a Physical Vapour Deposition-CoCrMo Biomedical Alloy. *Electrochimica Acta* **2015**, *180*, 96–103, doi:10.1016/j.electacta.2015.08.017.
81. Zhou, Y.; Hu, X.; Dou, C.; Liu, H.; Wang, S.; Shen, P. Structural Studies on Metal-Serum Albumin. IV. The Interaction of Zn(II), Cd(II) and Hg(II) with HSA and BSA. *Biophys Chem* **1992**, *42*, 201–211.
82. Debiemme-chouvy, C.; Haskouri, S.; Cachet, H. Study by XPS of the Chlorination of Proteins Aggregated onto Tin Dioxide during Electrochemical Production of Hypochlorous Acid. **2007**, *253*, 5506–5510, doi:10.1016/j.apsusc.2006.12.077.
83. Chen, W.; Wu, X.; Car, R. X-Ray Absorption Signatures of the Molecular Environment in Water and Ice. **2010**, *017802*, 1–4, doi:10.1103/PhysRevLett.105.017802.
84. Boonaert, C.J.P.; Dufre, Y.F.; Derclaye, S.R.; Rouxhet, P.G. Adhesion of Lactococcus Lactis to Model Substrata : Direct Study of the Interface. **2001**, *22*, 171–182.
85. Torres Bautista, B.E.; Carvalho, M.L.; Seyeux, A.; Zanna, S.; Cristiani, P.; Tribollet, B.; Marcus, P.; Frateur, I. Effect of Protein Adsorption on the Corrosion Behavior of 70Cu-30Ni Alloy in Artificial Seawater. *Bioelectrochemistry* **2014**, *97*, 34–42, doi:10.1016/j.bioelechem.2013.10.004.
86. Fears, K.P. Measuring the PK/PI of Biomolecules Using X-Ray Photoelectron Spectroscopy. *Analytical Chemistry* **2014**, *86*, 8526–8529, doi:10.1021/ac5020386.
87. Pradier, C.M.; Ka, F. Adsorption of Bovine Serum Albumin on Chromium and Molybdenum Surfaces Investigated by Fourier-Transform Infrared Reflection - Absorption Spectroscopy (FT-IRRAS) and X-Ray Photoelectron Spectroscopy. **2003**, 6766–6773, doi:10.1021/jp026365i.
88. Valero-Vidal, C.; Igual-Muñoz, A.; Olsson, C.-O.A.; Mischler, S. Adsorption of BSA on Passivated CoCrMo PVD Alloy: An EQCM and XPS Investigation. *Journal of The Electrochemical Society* **2014**, *161*, C294–C301, doi:10.1149/2.038406jes.
89. Frateur, I.; Lartundo-Rojas, L.; Méthivier, C.; Galtayries, A.; Marcus, P. Influence of Bovine Serum Albumin in Sulphuric Acid Aqueous Solution on the Corrosion and the Passivation of an Iron-Chromium Alloy. *Electrochimica Acta* **2006**, *51*, 1550–1557, doi:10.1016/j.electacta.2005.02.116.
90. Swaidan, A.; Borthakur, P.; Boruah, P.K.; Das, M.R.; Barras, A.; Hamieh, S.; Toufaily, J.; Hamieh, T.; Szunerits, S.; Boukherroub, R. A Facile Preparation of CuS-BSA Nanocomposite as Enzyme Mimics: Application for Selective and Sensitive Sensing of Cr(VI) Ions. *Sensors and Actuators, B: Chemical* **2019**, *294*, 253–262, doi:10.1016/j.snb.2019.05.052.
91. Sanner, M.F.; Olson, A.J.; Spehner, J.C. Reduced Surface: An Efficient Way to Compute Molecular Surfaces. *Biopolymers* **1996**, *38*, 305–320, doi:10.1002/(sici)1097-0282(199603)38:3<305::aid-bip4>3.0.co;2-y.
